# Assessing pricing and reimbursement policies for generic pharmaceuticals in the MENA region for improved efficiency, affordability and generic penetration

**DOI:** 10.1016/j.hpopen.2021.100045

**Published:** 2021-06-24

**Authors:** Bregtje W. Kamphuis, Panos Kanavos

**Affiliations:** Department of Health Policy, Medical Technology Research Group – LSE Health, London School of Economics and Political Science, UK

## Abstract

**Background:**

Healthcare systems in the Middle East & North Africa (MENA) region face challenges in healthcare service funding and delivery. Greater use of generics satisfies the objectives of macroeconomic efficiency and contributes to healthcare financial sustainability.

**Objective:**

To examine and critically appraise the key features of supply- and demand-side policies for generic pharmaceuticals in eleven MENA countries.

**Methods:**

This paper uses an analytical framework, informed by primary and secondary evidence, covering indicators across two themes: supply-side policies, including pricing, reimbursement and procurement policies for generic products, and proxy demand-side policies, particularly provisions for generic prescribing and generic substitution.

**Results:**

Evidence from primary and secondary sources suggests that price capping with and without managed competition and external reference pricing (ERP) are the principal generic pricing policies in the region, while reimbursement and procurement of generics rely on tendering. MENA countries focus their efforts predominantly on supply-side policies to reduce cost, whereas demand-side policies, such as generic prescribing and substitution policies, are rarely in place, and if they are, they are not mandatory in the majority of cases.

**Conclusion:**

There are significant opportunities for MENA countries to tailor their generic policies more closely to their individual health systems to improve efficiency and cost-effectiveness. Owing to the region’s inherent mistrust in generics, supply-side policies may be insufficient alone to achieve the countries’ efficiency and cost-effectiveness aims. The eleven MENA countries should consider a more holistic approach and pair supply-side with demand-side policies.

## Highlights

• Generic policies are key to realising macroeconomic and microeconomic efficiency objectives.
• Generic pricing policies should maximise price difference between generics and originators.
• Price capping and tendering should be used more aggressively to reduce prices.
• Mandatory generic prescribing and substitution mechanisms are often unused or voluntary.
• Appropriate demand-side policies are necessary given mistrust in generics across region.

## 1 Background

Health care systems in the Middle East & North Africa (MENA) region face challenges in the delivery of healthcare services due to a combination of a rising prevalence of non-communicable diseases, economic and fiscal pressures, and, in some countries, renewed focus on universal health insurance coverage which exerts more pressure on health care financing. Current total health expenditure in 2018 ranges between 2.49% (Qatar) and 8.35% (Lebanon) of Gross Domestic Product (GDP) across the MENA region, while government health spending ranges between 1.42% (Egypt) and 4.4% (Kuwait) of GDP [1], [2]. Average pharmaceutical spending as a percentage of health expenditure was 24.8% in 2016 across the MENA countries, compared to 17.4% across the OECD countries in the same year [3], [4], [13], [14], [5], [6], [7], [8], [9], [10], [11], [12]. In-patent pharmaceutical products accounted for an average of 63.3% of all prescription pharmaceutical sales across the region in 2016, while generic sales accounted for the remaining 36.7% [4], [5], [14], [6], [7], [8], [9], [10], [11], [12], [13]; countries like Jordan had a comparable performance to OECD countries in terms of their share of generic sales (58.3% of all prescription drug sales in 2017). In 2017, the volume share of generics in the total pharmaceutical market was reported to be around 52% in OECD countries [15] although similar data on the volume share of generics in the MENA region is not available. Most countries in the MENA region have fragmented healthcare systems and rely on funding from a combination of government expenditure, national health insurance schemes, and out-of-pocket spending. The gap between total and government health expenditure constitutes out-of-pocket spending; while most MENA countries claim they have comprehensive health insurance coverage, in practice incomplete coverage results in a substantial portion of pharmaceutical expenditure paid for out-of-pocket. 2018 data shows out-of-pocket spending as a percentage of current health expenditure ranged from 6% in Oman to 62% in Egypt [16].

Against the backdrop of a challenging healthcare environment, policy emphasis is often placed on health care cost minimisation and efficiency. Across the MENA countries, efforts to optimise healthcare costs have focused predominantly on controlling pharmaceutical costs given their significant contribution to overall health expenditure [17]. And while, generally, generic sales constitute a smaller proportion of total pharmaceutical sales than patented products across the region, policies for generic pharmaceuticals are key tools in attaining cost containment and efficiency objectives through the achievement of both low generic prices through supply-side methods, and high generic volumes through demand-side mechanisms. The importance of generic pharmaceuticals, as cheaper, high quality and cost-effective alternatives to off-patent originators becomes greater when accounting for the out-of-pocket burden for pharmaceuticals in the region.

While generic pharmaceuticals are of growing importance in the achievement of health system objectives and managing healthcare budgets in MENA countries, there are no studies assessing generic pharmaceutical policies in the region. In this paper, we address this gap in two ways. First, we develop an analytical framework to analyse generic pharmaceutical policies in the MENA region, with ten indicators pertaining to proxy demand- and supply-side policies. Second, we evaluate the impact of generic pharmaceutical policies in the eleven MENA countries against the objectives of cost-containment and efficiency.

## 2 Methods

In this paper, we apply a three-step methodological approach comprising secondary data collection through a comprehensive literature review, primary data collection from a variety of competent authorities and an analysis of the primary and secondary evidence collected utilising an analytical framework for generic policies in the eleven study countries.

### 2.1 Data extraction from secondary sources

A review of secondary sources was conducted through a comprehensive literature review on generic supply-side and demand-side policies and their possible impact in the eleven study countries: Algeria, Bahrain, Egypt, Jordan, Kuwait, Lebanon, Morocco, Oman, Qatar, the Kingdom of Saudi Arabia, and the United Arab Emirates (UAE). ProQuest, Web of Science, Medline, EconLit and CINAHL were searched using keywords related to pricing and reimbursement of generic pharmaceuticals and study country names based on an analytical framework (Table 1). The search strategy was limited to English language articles published between 1 January 2000 and 31 August 2020 and restricted the inclusion of documents to those with agreed upon keywords in their titles and abstracts. All documents were downloaded and imported to EndNote and duplicate references were removed. The articles were initially screened by the authors for relevance by title and abstract. Papers with relevant titles and/or abstract were downloaded and examined for relevance against the selected endpoints. Publications providing information on at least one of the endpoints were included, and the authors extracted the relevant information against each endpoint in an excel spreadsheet. A targeted search of official documents available through health ministries and/ or regulatory authorities’ websites in the countries of interest, the World Health Organization, the World Bank and other grey literature sources, including market sources, were also reviewed to identify further information on the endpoints. Additionally, a targeted Google search was performed, the results of which were imported in the same spreadsheet, where the relevant text was extracted and sorted against the study endpoints.

**Table 1:** Analytical framework and key endpoints for generic pharmaceutical policies in MENA region.

	**Key indicators**	**Endpoints**
**Supply-side policies**	A. Generic pricing policies	1. Pricing policy(ies) for generics: — Free pricing — Cost-plus pricing — Price capping — Price capping with managed entry — External Reference Pricing (ERP) — Free pricing — Cost-plus pricing — Combination or selection of lowest price from various methods — Other regulatory methods
		2. Existence of post patent expiry price change for off-patent originator pharmaceuticals
		3. Variation in generic pricing policies for local and imported generics
	B. Reimbursement and procurement policies	4. Reimbursement/procurement policy for generics: — Tendering — Internal Reference Pricing (IRP) (therapeutic reference pricing; molecular reference pricing; managed competition) — Formulary management
		5. Reimbursement of off-patent originator pharmaceuticals
		6. Variation in generic reimbursement policies for local and imported generics
**Proxy demand-side policies**	C. Generic prescribing	7. Existence of a (national) policy on generic prescribing
		8. Mandatory or non-mandatory generic prescribing
	D. Generic substitution	9. Existence of a (national) policy on generic substitution
		10. Mandatory or non-mandatory generic substitution

***Source***: The authors*.*

### 2.2 Primary data collection

Primary data was collected through a survey (May to September 2018) with experts and competent authorities from the eleven MENA countries. Survey questions were designed according to the salient features and performance indicators identified. We identified several stakeholders, including academic drug policy experts, experts from governmental agencies (including pharmacy departments, regulatory authorities, procurement agencies and insurance organisations) and industry executives, who were subsequently contacted with a request to assist with the completion of the survey. Combined responses (fusing expertise from different governmental agencies and departments) were received from nine countries (Algeria, Bahrain, Egypt, Jordan, Kuwait, Lebanon, Morocco, Qatar, and the UAE). Two countries (Saudi Arabia and Oman) did not return surveys. The lack of survey responses from Saudi Arabia was mitigated by the incorporation of the most recent pharmaceutical pricing policy, implemented in January 2021, into this paper.

A selection of survey respondents was invited to and participated in telephone interviews (n = 9) in which they were asked questions relating to two themes: (a) off-patent originator and generic price setting and pricing policies, and (b) reimbursement and coverage decisions relating to off-patent originators and generics. A semi-structured interview guide was set up for this purpose to ensure that local insights supplemented survey response data and data from the literature in order to further clarify and validate our findings on study country generic policies. The interview questions were designed in accordance with the analytical endpoints in Table 1. We coded the participant responses thematically to highlight key features and trends amongst the eleven study countries. Finally, in order to validate the original findings through face-to-face meetings with several experts from competent authorities, a further round of triangulation occurred through face-to-face interviews (n = 7) between one author (PK) and experts and decision-makers from the region (UAE, Saudi Arabia, Egypt, Lebanon, Jordan, Morocco and Bahrain) at the ISPOR conference in Dubai in September 2018. During these interviews it was possible to obtain further insights on the Saudi policy setting.

**Table 2:** Concepts in pricing and reimbursement of generic pharmaceuticals.

**Free pricing**	Prices are set by the manufacturer with no direct price control regulation applied. Indirect price control regulation may be in place, e.g. on reimbursement.
**Cost-plus pricing**	Prices are set by considering manufacturing costs and adding allowances for sales, promotional or marketing expenses, manufacturer profit margins, and charges and profit margins accrued in the supply chain to the production cost of a pharmaceutical product.
**Price capping**	Prices are set at a fixed percentage below the originator price at patent expiry.
**Price capping with managed entry**	Prices are set at a fixed percentage below the originator price, with additional specific percentage reductions depending on the sequence of market entry of each product.
**External Reference Pricing**	Prices are set by using the prices of the given medicine in one or several other countries to derive a benchmark or reference price.
**Combination or selection of lowest price from various methods**	Prices are set at the lowest price obtained from a single pricing method when reviewing resulting prices from a variety of pricing methods per product.
**Tendering**	A procedure using competitive bidding for the purchasing of medications. Suppliers bid for a particular contract with a specific quantity and price (and other criteria if this is permitted).
**Internal Reference Pricing**	A maximum level of reimbursement for a group of drugs is established as a benchmark or reference price. The drugs compared within a group can be identified at therapeutic level (therapeutic reference pricing) or at molecular level (molecular reference pricing).
**Formulary management**	A list of pharmaceutical drugs approved for reimbursement. A formulary can be positive (listing products that are reimbursed) or negative (listing products which are not reimbursed).

***Source***: The authors*.*

### 2.3 Analysis

The analytical framework considers both supply-side and demand-side policies for generic pharmaceuticals. For supply-side policies, the framework includes indicators on the type of generic pricing policies in use, price changes for off-patent originator, and variation in these policies for local and/or imported products. The supply-side component also takes generic reimbursement and procurement policies into account, including the type of reimbursement policy in place for generics and variation in these policies for local and/or imported products. For demand-side policies, the framework includes indicators on both generic prescribing and generic substitution, notably the existence of such a policy in each setting and whether the implementation of the policy is made mandatory. These endpoints are listed in Table 1.

Endpoints were used to perform a comparative mapping of pricing systems in the study countries and assess each country’s policies for generic pharmaceuticals. Table 2 provides an overview of key concepts and definitions. Literature on the impact of either supply- or demand-side policies was also reviewed. Based on the primary and secondary data we collected, generic pharmaceutical policies in the study countries were assessed on a country-by-country basis and for each endpoint.

## 3 Results

### 3.1 Generic pricing policies in the MENA region

In the MENA region, generic prices are often set through external price referencing (ERP) or price capping. Price capping is often applied in conjunction with managed competition, which sets out a scheme by which prices decrease based on order of market entry. Additionally, cost-plus pricing, and lowest price selection, together with other methods, are utilised by some of the eleven study countries. Table 3 summarises the generic pricing policies used by the countries.

**Table 3:** Pricing policies for generic pharmaceuticals in the MENA Region and implications for off-patent originators, 2018.

	Pricing policies for generic pharmaceuticals^1^							Off-patent originator pharmaceuticals	
	*Price capping*	*Price capping with managed competition*	*ERP*	*Free pricing*	*Cost-plus pricing*	*Combination or selection of lowest price from various methods*^4^	*Other regulatory methods*	*Does price decline post patent expiry?*	*If price declines, by how much?*
**Algeria**	✓^” α^	✘	✓^† α^	✘	✓^‡ α^	✘	✘	✓	–
**Bahrain**	✘	✓ ^α β^	✓ ^α^	✘	✘	✓ ^α^	✘	✓	20%
**Egypt**	✘	✓^”α β^	✘	✘	✘	✘	✘	–	–
**Jordan**	✓^α β^	✘	✓^† α β^^3^	✘	✘	✓^† α β^	✘	–	–
**Kuwait**	✘	✓ ^α^	✘	✘	✘	✘	✘	✓	20%
**Lebanon**	✓ ^β^	✘	✘	✘	✘	✘	✘	–	–
**Morocco**	✓	✓ ^β^	✓^*α^	✘	✘	✘	✘	✓	–
**Oman**	–	–	✓^~ β^	✘	✘	✘	✘	–	–
**Qatar**	✘	✓^~^	✘	✘	✘	✘	✓ ^α β^ profit controls	✓	20%^2^
**Saudi Arabia**	✘	✓^” β^	✘	✘	✘	✘	✘	✓ ^β^	25%
**UAE**	✓ ^β^	✓^β^^5^	✓^† β^^5^	✘	✘	✓ ^β^	✘	✓	20%

***Key:*** ✓ = Yes/used.

✘ = No/not used.

- = No evidence.

✓† = Applies only to imported generics.

✓‡ = Applies only to locally manufactured generics.

✓” = Different calculations/percentages for locally manufactured and imported generics.

✓* = solely applicable when the originator is not present in the home market

✓~ = only used for the private sector.

✓α = finding from primary data collection.

✓β = finding from secondary data collection.

Sources: The authors, based on findings of primary and secondary evidence.

***Note:***

1 Based on the results of primary data collection, whereas secondary sources, where used, reflect earlier years. There may be discrepancies in the application of some of the rules outlined in the table.

2 Prices decrease on registration of first generic.

3 ERP based on the price in the COO only.

4 Several countries identify the lowest price resultant from a selection of methods. These methods may include price capping, price capping with managed competition, and ERP, as well as other sources or methods for pricing.

5 The UAE uses price capping with managed competition and ERP for imported or partially locally manufactured products.

#### 3.1.1 Price capping

Price capping is used in Algeria and Lebanon for both imported and locally manufactured generics, and in Jordan and the UAE for wholly locally manufactured generics. Prices for locally manufactured products are set between 50 and 60% (partially locally manufactured or imported generics in the UAE), 70% (Lebanon; fully locally manufactured generics in the UAE; cases of contract manufacturing in Jordan; imported generics in Algeria) to 80% (Algeria, Jordan) of the originator price [4], [18], [19], [20], [21], [22], [23]. In Lebanon, the price to consumers for generics is 30% less than originator brands [24], [25], though two exceptions exist: a) if no originator is registered prices are reviewed three months after a third generic is registered and an average price is set, and b) if the price of the originator changes, the generic price will be set at a rate half of the originator [24].

Morocco operates a different price capping mechanism: prices of locally manufactured or imported generics are set using a minimum rate of reduction of the originator price, but the percentage reduction is set according to the originator’s price: ranging from 0%, where the originator price is under 15 dirham (approximately 4 US dollars), to 50%, in cases where originator prices are over 300 dirham (approximately 33 US dollars) [18].

#### 3.1.2 Price capping with managed competition

A price capping system with elements of managed competition based on sequence of entry is utilised for pricing generics in Bahrain, Egypt, Kuwait, Morocco, Qatar, and Saudi Arabia, and for partially locally produced or imported products in the UAE [5], [18], [21], [23], [24], [31], [32], [33], [34], [35], [26], [27], [28], [29], [30]. Across these countries, the price of the first entrant(s) is set between 15% and 40% lower than the originator product. In Bahrain, Kuwait, Qatar, and Saudi Arabia the price set for the first generic is based on an amended price of the originator drug after loss of exclusivity. The UAE sets a percentage decrease for generic pharmaceuticals based on the cost, insurance and freight (CIF) price approved for the branded product before any deductions [23]. It is unclear whether pricing mechanisms in Egypt and Morocco rely on the original price of the in-patent product or the price after loss of exclusivity. In Bahrain, Kuwait, Saudi Arabia, and the UAE a set of subsequent entrants to the market experience a further stepwise decline of between 5 and 10% after which prices should remain lower or equal to the price of the final entrant. In the Qatari private sector, the second generic is priced 10% less than the first, the third generic is 10% less than the second, and all subsequent generics are 10% less than the third generic [24], [25]. Limited generics are found in the Qatari public sector, which are reported to have no price difference with originator brands [24].

Egypt and Morocco employ a slightly modified managed competition structure. The Egyptian pricing regime groups the first five generics entering the market into a cluster all priced at 35% below the originator’s price, after which all subsequent entrants are priced 40% below the originator’s price [18], [32], [33], [35]. In Morocco, pricing depends on the retail price for the originator: where the originator price is lower or equal to 250 Moroccan dirhams, the first nine generics are priced 50% lower. Where the originator price is higher than 250 Moroccan dirhams, the first nine generics are priced up to 50% less than the price of the originator. All generics that follow are priced 20% lower than the first nine [5], [29], [30]

Capping percentages may be adjusted depending on the source and type of product in some settings. For example, in Egypt, differentiation is made between imported generics (priced 35% below originator price for first five entrants, with subsequent entrants at 40% below originator price), high-technology generics (if from a reference country set at 30% below the originator’s price, and if not from a reference country at 35%), and locally manufactured generics (priced 60 to 75% below originator prices with a subsequent 10% reduction) [18], [21], [32], [33], [35].

#### 3.1.3 External reference pricing

ERP is used as a pricing practice for generics in Algeria, Bahrain, Jordan, Morocco, Oman, Saudi Arabia, and the UAE, though applied predominantly in restricted cases: only for imported generics (Algeria, Jordan, UAE), partially locally produced pharmaceuticals (UAE), only for locally manufactured generics where the originator is not available in the local market (Saudi Arabia), for drugs in the private sector (Oman), and in cases where no originator exists in the market (Morocco) [18], [20], [23], [30], [36], [37], [38], [39], [40]. Moroccan authorities calculate a maximum reference price for generic products by applying a reduction rate to a manufacturer’s selling price set in six countries plus the country-of-origin (COO) [18].

#### 3.1.4 Cost-plus pricing

Algeria is the only country to apply cost-plus pricing, though the practice is restricted to locally manufactured generics [18].

#### 3.1.5 Selection of the lowest price from various methods

Bahrain, Jordan and the UAE apply multiple pricing methods from which the lowest price is selected to price pharmaceutical products. The use of this method is designated only for specific cases in Jordan and the UAE: imported generics (the UAE), imported generics with a locally registered generic equivalent (Jordan), and partially locally manufactured generics (the UAE).

In Bahrain, both ERP and price capping with managed competition are used to obtain the lowest price, in addition to the ex-factory price in the COO plus the cost of insurance and shipping and prices of drugs in the same therapeutic category and/or with the same therapeutic effect [18].

In Jordan prices for imported generics with a locally registered equivalent are determined as the lowest price out of a price capping system using 80% of the originator price, the export price to the Saudi market and the price in the COO [18], [20]. If there is no locally registered equivalent, imported products are priced as originators [18].

In the UAE prices for generics result from the selection of the lowest price from a price capping with managed competition system, setting prices at 60% of the CIF price approved for the innovator before deduction (at 50% for the second and 40% for the third entrant), ERP, which takes the median CIF price across reference countries, the ex-factory price in the COO, with an additional 20% and the CIF price proposed by the manufacturer [23]. For products to be registered as generics but with no originator or generic equivalent registered in the UAE, the product is priced according to the originator pricing criteria: the lowest price between the ex-factory price, import price proposed by the manufacturer, and the median CIF price in reference countries [23].

Pharmaceutical pricing in Saudi Arabia relies on price capping with managed competition, though the pricing guidelines set out a number of further criteria to take into account: therapeutic value; prices of registered alternative treatments in Saudi Arabia; pharmacoeconomic studies; ex-factory price in the currency of the country of the manufacturer; wholesale price in the currency of the COO; the price submitted by the manufacturer for Saudi Arabia; the ex-factory price in countries where the product is marketed; the public price in the COO and countries where the drug is marketed; and the price of the drug in the approved reference countries (which may differ from countries where the product is marketed) [34]. It is not clear in what capacity these additional criteria are used: whether the lowest price is selected, or whether these criteria are only supplementary guidance.

#### 3.1.6 Other price-regulation methods

Qatar also relies on other criteria and methods to price imported generics. Qatar considers CIF prices and employs a profit control mechanism [18]. Following the dissolution of Law No.7 in 1990, pharmaceutical retail price control by the government enables importers to determine their own prices [41].

#### 3.1.7 Post-patent expiry price changes for originator pharmaceuticals

The originator price declines post patent expiry in six of the MENA countries (Algeria, Bahrain, Kuwait, Morocco, Saudi Arabia, and the UAE). For generic pricing policies, and price capping mechanisms in particular, to work effectively off-patent prices should decrease on expiry of the patent: evidence indicates that the originator price decreases by 20% post-patent expiry in Bahrain, Kuwait and the UAE and 25% in Saudi Arabia [18], [23], [26], [28], [34]. In Qatar the originator price is reduced by 20% on registration of the first generic in the Qatari market [24]. The Saudi Arabian pricing guidelines also stipulate that any re-pricing of the originator product during the first renewal of registration after a generic product is registered and marketed, will see accompanying re-pricing of the generic product(s) to ensure the difference between the price of the originator and generic product(s) is not less than 10% [34]. No information was found on whether off-patent prices decline post-patent expiry in Algeria, Egypt, Jordan, Lebanon, or Oman.

### 3.2 Reimbursement policies and procurement strategies

Many of the countries in the MENA region have fragmented reimbursement systems with multiple actors involved in the purchasing of pharmaceuticals, delivery of healthcare, and reimbursement mechanisms. Table 4 provides an overview of the main methods for procurement and reimbursement for off-patent originators and generic pharmaceuticals in the MENA region.

**Table 4:** Reimbursement and procurement policies for off-patent and generic medicines, 2018*.

		**IRP**^1^**(molecular)**	**IRP (therapeutic)**	**IRP (managed competition)**	**ERP**	**Tendering**	**Formulary management**	**Bio-equivalence**
**Algeria**	Off-patent	✓ ^α^	✓ ^α^	✘	✓ ^α -^	✓ ^α^^2^	✓ ^α^^2^	N/A
	Generic	✓ ^α^	✓ ^α^	✘	✘	✓ ^α^^2^	✓ ^α^^2^	✘
**Bahrain**	Off-patent	–	–	–	–	–	–	N/A
	Generic	–	–	–	–	–	–	–
**Egypt**	Off-patent	✘	✘	✘	✘	✓ ^α^	✘	N/A
	Generic	✘	✘	✘	✘	✓ ^α^	✘	✘
**Jordan**	Off-patent	–	–	–	–	–	–	N/A
	Generic	–	–	–	–	✓ ^β^	–	–
**Kuwait**	Off-patent	✘	✘	✘	✘	✓ ^α^	✘	N/A
	Generic	✘	✘	✘	✘	✓ ^α^	✘	✘
**Lebanon**	Off-patent	✘	✘	✘	✘	✓ ^α^	✓	N/A
	Generic	✓ ^α^	✓ ^α^	✓ ^α^	✘	✓ ^α^	✓ ^α^	✓ ^α^
**Morocco**	Off-patent	✓ ^α^	✘	✘	✘	✓ ^α^	✘	N/A
	Generic	✓ ^α^	✘	✘	✘	✓ ^α^	✘	✘
**Oman**	Off-patent	–	–	–	–	–	–	N/A
	Generic	–	–	–	–	–	–	–
**Qatar**	Off-patent	✓ ^α^	✘	✘	✓- ^α -^	✓ ^α^	✓ ^α^	N/A
	Generic	✓ ^α^	✘	✘	✘	✓ ^α^	✓ ^α^	✓ ^α^
**Saudi Arabia**	Off-patent	✘	✘	✘	✘	✓ ^β^	✘	N/A
	Generic	✓ ^β †^	✓ ^β †^	✘	✘	✓ ^β^	✘	✘
**United Arab Emirates**	Off-patent	✘	✘	✘	✘	✓ ^α^	✘	N/A
	Generic	✘	✘	✘	✘	✓ ^α^	✘	✘

***Key:*** ✓= yes / used.

✓- = used as a reference price for guidance.

✓* = only used in hospitals, not at national level.

✓α = finding from primary data collection.

✓β = finding from secondary data collection.

✘ = no / not used.

- = no evidence.

N/A: not applicable.

Source: The authors, based on findings of primary and secondary evidence.

***Notes****:* * Applies to all medicines whether outpatient or in-patient; if relevant to out- or in-patient, a qualification is made where appropriate.

1 Internal reference pricing.

2 Only used in hospitals, not at out-patient level.

#### 3.2.1 Reimbursement of off-patent originator pharmaceuticals

Tendering is the most prevalent mechanism for the procurement of *off-patent* pharmaceuticals and is used in eight countries: Algeria, Egypt, Kuwait, Lebanon, Morocco, Qatar, Saudi Arabia, and the UAE [18], [28], [42]. Tendering is used in combination with other mechanisms for the reimbursement of off-patent pharmaceuticals in several countries. One such mechanism is internal reference pricing (IRP) (used in Algeria, Morocco and Qatar), while another is whileformulary management (Algeria, Lebanon and Qatar) [18]. Prices resulting from ERP calculations are used as guidance or negotiation starting points for off-patent products in Algeria and Qatar [18]. In Algeria tendering and formulary management for generics are only used within hospitals, and IRP is used to set the reference tariff for reimbursement [18]. No evidence was found on the reimbursement of off-patent pharmaceuticals for Bahrain, Jordan and Oman.

#### 3.2.2 Reimbursement of generic pharmaceuticals

Tendering is the predominant mechanism for the procurement of *generic* pharmaceuticals, used in nine countries (Algeria, Egypt, Jordan, Kuwait, Lebanon, Morocco, Qatar, Saudi Arabia, and the UAE) [18], [28], [42]. In Jordan, public sector procurement is performed through annual tenders issued in the generic name of the pharmaceuticals or therapeutic groups jointly issued by four governmental parties [37], [43]. In Algeria tendering and formulary management for generics are only used within hospitals [18].

In Algeria, Egypt, Kuwait, Morocco, Saudi Arabia and the UAE, generics are reimbursed according to the same system as off-patent pharmaceuticals. Although notably, reimbursed prices for generics in the UAE vary according to the country of manufacture: generics manufactured in the UAE or other members of the Gulf Cooperation Council (GCC) are reimbursed at 20% below the originator price, while imported generics are priced at 30% below the originator price [36]. Egypt has a policy (the ‘box’ system) which restricts the market to a maximum of 12 products per pharmaceutical composition: one branded/innovator product and eleven generic products [18], [30], [35]. The eleven generics are usually made up of one imported generic compared to ten locally produced generics [18], [30], [35]. The box system aims to encourage local investment in manufacturing, import substitution and direct market resources towards neglected pharmaceutical products [30].

IRP is sometimes used for pricing of imported generics in Saudi Arabia. The pricing regulation states that such a price cannot exceed the lowest price of similar registered products [21]. Algeria may also rely on either molecular or therapeutic reference pricing for the reimbursement of generic pharmaceuticals [18].

Lebanon and Qatar adopt slightly different reimbursement strategies for generics compared with off-patent originator pharmaceuticals. In Lebanon tendering and formulary management are dominant, but IRP and bioequivalence may also be used [18]. In Qatar reimbursement is achieved through a combination of different measures, involving tendering, formulary management and IRP. In the context of IRP, the grouping of generic pharmaceuticals is based on molecular reference pricing [18].

Algeria may also rely on different reimbursement methods in specific cases: for example, where an originator with the same active substance received a prior positive decision and is already reimbursed, generic products are automatically reimbursed according to their fixed price, and where the generic is the first entrant to the market, budget impact criteria and ERP are used to determine reimbursement [18].

No evidence was found on the reimbursement of generic pharmaceuticals for Bahrain and Oman.

Public procurement in the region sometimes gives preferential treatment, including price advantages, to local manufacturers’ products (this practice is seen in Bahrain, Egypt, Jordan, Kuwait, Lebanon, Morocco, Qatar, and Saudi Arabia) [9], [11], [42], [44], [45], [46], [47], [48]. An example of such treatment in Egypt and Saudi Arabia is how locally manufactured products can win a tender even if their submitted price is up to 10% higher than the lowest submitted price [18]. Algeria imposes a ban on imported pharmaceuticals if locally produced alternatives are available in the country, with reports suggesting the policy is applied in an inconsistent manner [49].

#### 3.2.3 Generic prescribing and substitution

Table 5 provides an overview of the generic prescribing and substitution policies in place in the MENA countries. Policies formalizing generic prescribing as mandatory exist only in four of the study countries: Jordan, Oman, Qatar, and the UAE [38], [39], [50], [51], [52]. Out of all the countries with mandatory generic prescribing policies, no country has implemented an electronic system to manage the practice [18] and, as a result, it is uncertain whether generic prescribing is actively practiced or encouraged. Algeria, Lebanon, and Morocco have policies encouraging generic prescribing, while in Bahrain and Saudi Arabia generic prescribing is allowed but it is unclear whether policies mandating this exist and whether they have been implemented [18], [42], [48], [53].

**Table 5:** Generic prescribing and substitution policies in the MENA region*.

	**Generic prescribing**			**Generic dispensing/substitution**	
	Is there a generic prescribing policy in place?	Is generic prescribing mandatory or encouraged within existing policy? 2	For mandatory generic prescribing policies, is there an IT system? *(not applicable (n/a) for non-mandatory systems)*	Is there a generic substitution policy in place?^2^	Is generic substitution mandatory or encouraged?^2^
**Algeria**	✓ ^α^	Not mandatory ^α^	n/a	✓ ^α^	Not mandatory ^α^
**Bahrain**	- ^β^	Not mandatory ^β^	n/a	✓ ^β^	Not mandatory ^β^
**Egypt**	✘ ^α^^1^	✘ ^α^	n/a	✓ ^α^	Not mandatory ^α^
**Jordan**	✓ ^β^	Mandatory ^β^^3^	No	✓ ^β^	Not mandatory ^β^
**Kuwait**	✘	✘	n/a	✓ ^β^	Not mandatory ^β ‡^
**Lebanon**	✓^α β^	Not mandatory ^α β^	n/a	✓^α β^	Not mandatory ^α β^
**Morocco**	✓ ^α^	Not mandatory ^α^	n/a	–	–
**Oman**	✓ ^β^	Mandatory ^β ‡ 3^	No	✓ ^β^	Not mandatory ^β ‡^
**Qatar**	✓ ^β^	Mandatory ^β^^3^	No	✘ ^β^	✘ ^β^
**Saudi Arabia**	- ^β^	Not mandatory ^β^	n/a	✓ ^β^	Not mandatory ^β^
**UAE**	✓^α β^	Mandatory^‡ α β^^3^	No	✓^α β^	Not mandatory ^α β^

***Note:*** * The results in this table capture what official policies may suggest; in practice, generic prescribing may be overridden by prescribers suggesting a brand. More importantly, both generic prescribing and substitution policies are poorly implemented as trust on generics is often problematic due to likely substandard or counterfeit generics or their perception as such.

- : no information or data found. Where it is used for a non-mandatory system, it was unclear from the data whether a specific policy was in place or it is allowed by virtue of no regulation existing.

‡: information limited to the public sector.

✓^α^ = finding from primary data collection.

✓^β^ = finding from secondary data collection.

***Source***: The authors, based on findings of primary and secondary evidence.

1 There may not be an explicit generic prescribing policy in place, nevertheless, there is a generics policy in place through the Egyptian ‘box’ system.

2 Both columns reflect current policies as stated in legislation. However, due to the likelihood of poor quality products entering the market in many cases, it is not known how widely these policies are implemented in practice.

3 Not known how widely implemented this is.

Most countries in the MENA region have a generic substitution policy in place, except for Qatar [18], [24], [51]. However, in no country is generic substitution mandatory; Algeria, Egypt, Jordan, Kuwait, Lebanon, Oman, Saudi Arabia and the UAE only have policies or legislation which encourage or allow the practice [28], [38], [39], [42], [47], [48], [52], [53], [54], [55]. Jordanian legislation does not allow for generic substitution [43], [50]. No data was found for Morocco, though other practices observed in Morocco include efforts to encourage insurers to pay for the cheapest generic drug(s), requiring the patient to pay the difference if they choose to use a branded product [5], [29].

### 3.3 Discussion and policy implications

Pharmaceutical affordability is the result of a complex interdependency of pricing frameworks, coverage levels and decisions, healthcare system financing, trust in generic pharmaceuticals, and the out-of-pocket burden, as well as inexorably linked to the way that pharmaceuticals are reimbursed, and the methods used to ensure that reimbursed prices are affordable. Pharmaceutical affordability is an issue of varying importance across the MENA region: interviewees reported it is not considered a key issue in the Algerian or Qatari markets, though it is considered problematic in Lebanon, Egypt, Morocco, and the UAE, and in Jordan depending on therapeutic class [18].

Generic pricing and reimbursement policies vary in much of the MENA region for locally manufactured and imported products, the underlying expectation being that preferential treatment of locally manufactured generics will stimulate national manufacturing capabilities. Support provided to local manufacturers includes predominantly discriminatory reimbursement practices favouring local over multinational manufacturers (seen in Algeria, Bahrain, Egypt, Jordan, Kuwait, Lebanon, Morocco, Qatar, and Saudi Arabia), and generic pricing policies to encourage local production and provide more beneficial pricing arrangements to local over imported generics (seen in Algeria, Egypt, Saudi Arabia, and the UAE) [18], [30]. Affordability concerns may emerge when these variations are used in combination with certain pricing options, such as price capping. An example arises in Jordan, where the design and application of the pricing policy are considered amongst the reasons pharmaceutical products in the country are unaffordable. This is as the pricing system enables local manufacturers to price their generic products as high as 80% of the originator price [20] in a system where local manufacturers may be more inclined to demand maximum prices because they rely on export markets where the COO price plays a significant role and the Jordanian market may present limited demand.

In general, off-patent and generic pharmaceutical reimbursement policies may be aligned to health system cost minimisation objectives, they may not necessarily achieve lower prices. This may also be the case in the MENA region due to two reasons: first, the study countries may procure more expensive innovative pharmaceuticals over cheaper, generic alternatives for reasons such as insufficient cheaper pharmaceuticals (Saudi Arabia), inadequate procurement of low-priced generics (Egypt, Jordan, Kuwait, Lebanon, and the UAE), unavailability of generics due to non-price factors (Egypt, Jordan, Kuwait, Lebanon, and the UAE), or unnecessary reliance on some innovator brands (Kuwait) [56], [57], [58]. Second, several of the MENA countries do not always achieve low prices for procured generics. The public sectors in Morocco, Lebanon and Kuwait often procure generics at a higher price than other countries in the Middle East [46], [59]. A study of 15 generic pharmaceuticals suggested average public sector procurement prices in Kuwait were 10% higher than international reference prices, with other studies suggesting lowest priced generic prices were 39% higher that the international reference price [8], [60]. However, conflicting information exists on prices in some settings: while some studies report higher prices in Jordan, others report the Jordanian system procures at prices which are reasonable compared to international reference prices [46], [61]. Lack of alignment and appropriate implementation of generic policies may also affect prices; in Bahrain, a policy for improved generic procurement in the public sector may have had an impact on overall prices [7].

Key design choices in pricing and reimbursement policies also impact generic medicines’ availability. Public sector procurement in Jordan was performed through independent annual tenders by four separate governmental parties prior to the introduction of the Joint Procurement Department and this previous system may have resulted in lower pharmaceutical availability and increased pharmaceutical expenditure because it encouraged double purchasing, whereby the government pays for more than one public health organisation purchasing the same pharmaceutical in the same year at distinct prices [20], [37]. In Egypt, the depreciation of the Egyptian pound resulted in shortages of several pharmaceutical products, including locally manufactured generics, attributed to currency pressures making raw materials more expensive and decreases in local manufacturer profits [6]. Similarly, the Algerian import ban on products which have a locally manufactured equivalent may be resulting in shortages of pharmaceutical products [49].

These findings suggest that current pricing and reimbursement policies should be reviewed based on their impact on affordability and availability in specific country contexts. Additionally, it is not unusual to find multiple pricing or reimbursement policies in place at a given time, as several countries rely on the results of multiple methods to identify list prices and procure at prices that arre better than the list. This may be a consequence of the varied geographic sources of pharmaceuticals in the region, creating a manner in which to ensure there is a pricing mechanism for which data is available. Other countries are unclear in how different pricing systems exist simultaneously, such as the use of price capping and price capping with managed competition in Morocco. Systems may be overly complex, resulting in difficulties in assessing true impact on price levels and patient access.

Countries in the region focus efforts predominantly on supply-side policies, and not on demand-side policies aimed at promoting the use and uptake of generic pharmaceuticals. Prescribing and dispensing behaviour are considered essential in encouraging generic uptake. There is an effort towards implementing mandatory generic prescribing in the region, currently in place in four study countries (Jordan, Oman, Qatar, and the UAE), while the practice is "encouraged" in two other study countries (Algeria and Lebanon). None of the study countries had implemented an electronic system to manage mandatory generic prescribing until the completion of this research, indicating that these systems may struggle to enforce such a requirement.

Generic substitution is not widely required in the MENA region: while most countries in the region have a generic substitution policy, it has only been made mandatory practice in Jordan. While permissive generic substitution allows pharmacists to dispense a generic alternative where they see fit, penetration and uptake is dependent on the willingness of pharmacists to do so. Mandatory generic substitution policies ensure generic uptake is as high as possible. Other options encouraging the use of generics may be to accompany permissive generic substitution policies with efforts at engaging and educating pharmacists on this topic. This trend is also linked to the relative lack of compulsion in the use of generic pharmaceuticals – where possible - in order to improve overall affordability for the health care system and patients.

Demand-side policies are key in managing the uptake of generic products in circumstances where brand loyalty or other preference-based behaviours may prevail, and, ultimately, can contribute to containing pharmaceutical expenditure. Table 5 depicts policies and practices in the region that may be included in national legislation, however, it is unknown how widely and effectively these are implemented. A large number of countries in the region have pharmaceutical consumption which is predominantly focused on branded products [7], [8], [62], [63], [13], [28], [50], [54], [56], [9], [10], [11] highlighting two key concerns with generics, particularly certain imported products: first, there is often a lack of trust in generics amongst prescribers and consumers/patients due to the possibility of substandard and/or counterfeit products entering the supply chain; and, second, partly as a result of the deficit in trust, prescribers are in a position to make suggestions to consumers on brands which, in their view, meet those quality standards. Overall, it appears that significant effort needs to be made for generics to be accepted by several of the countries in the region, particularly in terms of improving quality standards. That also means improvements in regulatory capacity and oversight.

The ideal policy for generic pharmaceuticals balances strong supply-side measures, which look to encourage low prices for the system and/or consumers, with essential demand-side policies, which ensure uptake of generic policies is as widespread as is necessary. Striking this balance is contingent on appropriate reflection of the country-specific context, including institutional, cultural and/or behavioural factors.

There are several solutions to the issues related to the pricing and reimbursement of off-patent originator and generic pharmaceuticals. First, adjustments could be made to increase generic procurement by governments for public sectors, where opportunities exist. This can favourably impact both prices and overall spending on pharmaceuticals. Second, to ensure pharmaceutical availability is not negatively impacted by tenders in fragmented healthcare systems, countries in the region could look to the solution adopted by Jordan, where joint procurement was introduced to prevent different public health institutions from buying the same pharmaceutical at different prices and which was found to lead to shortages and higher pharmaceutical spending [37]. Third, affordability could be improved by action through alternative channels, whether these are non-governmental organization activities or broader government action on pharmaceutical policy. Examples include the Lalla Salma Foundation in Morocco, which improved access to innovative pharmaceuticals for poor patients via a memorandum with pharmaceutical manufacturers to enable innovative oncological drug access [64], or the approval of several generic pharmaceuticals as part of the Moroccan National Drug and Pharmaceutical Policy directed at delivering affordable pharmaceutical products in 2017 [5]. In other instances, countries have resorted to price cuts to improve affordability. For example, the Lebanese government reduced the prices of 30 branded and 60 generic pharmaceuticals in 2015. This action was taken to increase access, though, despite these price reductions, many patients continue to struggle to afford medicines [9], [65]. Lastly, pricing policies could be reviewed for their balance in achieving both industrial and affordability aims. For example, the Jordanian pricing policy promotes competition between originators and generics, but not between different generics [20], [52]. Policy change could center on the broader supply chain, including distribution and generic manufacturing.

Even with appropriate pricing and reimbursement policy mechanisms, it is key for generic uptake to be managed correctly through prescribing and dispensing. Countries in the MENA region could organise action in these areas by creating national legislation for mandatory generic prescribing and substitution, designing policy interventions or other mechanisms aimed at improving generic prescribing and substitution, tracking the implementation of these policies, improving quality standards and measures to avoid sub-standard products on the market, and, where quality is sufficient, implementing efforts at promoting the use of and trust in generic products as appropriate substitutions for branded products for both healthcare professionals and patients.

Our analysis is not without limitations. Evidence on and analysis of the impact of pricing, reimbursement and procurement policies for generic pharmaceuticals in the MENA region is sparse and inconsistent. This study relies on a combination of secondary sources and a small sample of primary evidence to validate and fill evidence gaps, though evidence often remains anecdotal and is frequently confusing because of two important elements in the MENA region: the first is the incomplete coverage (including prescription drug coverage) by many health care systems, which effectively means that in many of the study countries there is a significant out-of-pocket element for health care services and, particularly, pharmaceuticals, while the second is inadequate distinction in the pharmaceutical market between originator pharmaceuticals and generics, and their respective impact. These two elements often lead to unclear or contradictory conclusions. Additionally, this study reveals a multiplicity of criteria used in pricing, resulting in difficulties in identifying a single or unique method used for the pricing of generic medicines: some countries rely on several methods from which to select a lowest price for a given product, while for others, multiple methods are referred to but with no clear indication of how these systems co-exist. The type of generic product may play a role in this context (e.g. branded vs. unbranded) as do national policies promoting and supporting local manufacturing.

## 4 Conclusion

Many of the countries in the MENA region have fragmented reimbursement systems with multiple actors involved in the purchasing of generic pharmaceuticals, delivery of healthcare, and reimbursement mechanisms. Pharmaceutical policies can impact the price levels achieved and affect the availability and affordability of off-patent originators and generic pharmaceuticals if well-balanced. However, countries in the MENA region focus efforts almost exclusively on supply-side policies to reduce cost and not on demand-side policies. There are significant opportunities for the study countries to tailor their generic policies more closely to their individual health systems to improve efficiency, cost-effectiveness and rational use. Countries should focus on increasing public sector procurement and designing pricing policies to achieve both industrial policy and health system efficiency and affordability aims. Countries should also aim to develop, implement and enforce policies for prescribing and dispensing generic pharmaceuticals to improve generic uptake. Given the region’s inherent mistrust in generics, supply-side policies focusing on pricing and reimbursement may be insufficient to achieve efficiency and cost-effectiveness aims and any reform should be paired with appropriate demand-side policies pertaining to generic prescribing and substitution coupled with a strengthening of regulatory capacity if needed.

## CRediT authorship contribution statement

**Bregtje W. Kamphuis:** Methodology, Investigation, Formal analysis, Writing - original draft. **Panos Kanavos:** Conceptualization, Methodology, Formal analysis, Supervision, Writing - review & editing.

## Declarations of Competing Interest

This research is in part based on a wider study, ‘Pharmaceutical pricing and reimbursement in the Middle East and North Africa region: A mapping of the current landscape and options for the future’, conducted by the authors. This wider study was funded by the Pharmaceutical Research and Manufacturers of America. The sponsor was not involved in the study design; collection, analysis, and interpretation of data; or writing of the report and publication of the study results.

## References

[1] World Bank. Domestic general government health expenditure (% of GDP), 2018 [Internet]. World Bank DataBank: World Development Indicators. 2021 [cited 2021 Mar 4]. Available from: https://databank.worldbank.org/source/world-development-indicators#

[2] World Bank. Current health expenditure (% of GDP), 2018 [Internet]. World Bank DataBank: World Development Indicators. 2021 [cited 2021 Mar 4]. Available from: https://databank.worldbank.org/source/world-development-indicators#

[3] OECD. Pharmaceutical spending (indicator), 2016. OECD Data; 2021.

[4] Business Monitor International (BMI). Jordan: Pharmaceuticals & Healthcare Report Q1 2018. Industry Report & Forecasts Series. London (UK); 2017.

[5] Business Monitor International (BMI). Morocco: Pharmaceuticals & Healthcare Report Q1 2018. Industry Report & Forecasts Series. London (UK); 2017.

[6] Business Monitor International (BMI). Egypt: Pharmaceuticals & Healthcare Report Q1 2018. Industry Report & Forecasts Series. London (UK); 2017.

[7] Business Monitor International (BMI). Bahrain: Pharmaceuticals & Healthcare Report Q1 2018. Industry Report & Forecasts Series. London (UK); 2017.

[8] Business Monitor International (BMI). Kuwait: Pharmaceuticals & Healthcare Report Q1 2018. Industry Report & Forecasts Series. London (UK); 2017.

[9] Business Monitor International (BMI). Lebanon: Pharmaceuticals & Healthcare Report Q1 2018. Industry Report & Forecasts Series. London (UK); 2017.

[10] Business Monitor International (BMI). Oman: Pharmaceuticals & Healthcare Report Q1 2018. Industry Report & Forecasts Series. London (UK); 2017.

[11] Business Monitor International (BMI). Qatar: Pharmaceuticals & Healthcare Report Q1 2018. Industry Report & Forecasts Series. London (UK); 2017.

[12] Business Monitor International (BMI). Saudi Arabia: Pharmaceuticals & Healthcare Report Q1 2018. Industry Report & Forecasts Series. London (UK); 2017.

[13] Business Monitor International (BMI). United Arab Emirates: Pharmaceuticals & Healthcare Report Q1 2018. Industry Report & Forecasts Series. London (UK); 2017.

[14] Business Monitor International (BMI). Algeria: Pharmaceuticals & Healthcare Report Q1 2018. Industry Report & Forecasts Series. London (UK).

[15] OECD. Health at a Glance 2019: OECD Indicators. Paris; 2019.

[16] World Health Organization. Out-of-pocket (OOPS) as % of Current Health Expenditure (CHE), 2018 [Internet]. Global Health Expenditure Database. 2021 [cited 2021 Mar 4]. Available from: https://apps.who.int/nha/database/Select/Indicators/en

[17] Kanavos P, Kamphuis BW, Fontrier A-M, Colville Parkin G, Saleh S, Akhras KS. Pricing of in-patent pharmaceuticals in the Middle East and North Africa: Is external reference pricing implemented optimally? Health Policy (New York) [Internet]. 2020;124(12):1297–309. Available from: https://www.sciencedirect.com/science/article/pii/S0168851020301998 10.1016/j.healthpol.2020.07.017 32962876

[18] Interviews conducted with experts from Algeria, Bahrain, Egypt, Jordan, Kuwait, Morocco, Lebanon, Qatar, and the UAE. P. Kanavos and V. Tzouma. [Interview]. Unpublished work.

[19] Business Monitor International (BMI). Jordan: Pharmaceuticals & Healthcare Report Q1 2017. Industry Report & Forecasts Series. London (UK); 2016.

[20] El-Dahiyat F, Curley L. Pharmaceutical Policy in Jordan. In: Babar Z-D, editor. Pharmaceutical Policy in Countries with Developing Healthcare Systems [Internet]. Cham, SWITZERLAND: Springer International Publishing AG; 2017. Available from: http://ebookcentral.proquest.com/lib/londonschoolecons/detail.action?docID=4831867

[21] Qarain M, Tabbaa T, Goussous R. Assessment of the current pricing policy on the pharmaceutical sector; 2009.

[22] Soualmi R, Bouabidi A, Djahdou Z, Ziouani S, Kaddar M, Mansouri K. Drug pricing and reimbursement processes in Algeria. Value Heal. 2016;19(7):PA448-A449.

[23] United Arab Emirates Ministry of Health & Prevention. Pricing Guidelines; 2018.

[24] Abdel Rida N, Mohamed Ibrahim MI, Babar Z-U-D. Pharmaceutical pricing policies in Qatar and Lebanon: narrative review and document analysis. J Pharm Heal Serv Res [Internet]. 2019 Sep 1;10(3):277–87. Available from: https://doi.org/10.1111/jphs.12304

[25] Abdel Rida N, Mohamed Ibrahim MI, Babar ZUD. Relationship between pharmaceutical pricing strategies with price, availability, and affordability of cardiovascular disease medicines: surveys in Qatar and Lebanon. BMC Health Serv Res [Internet]. 2019;19(1):973. Available from: https://doi.org/10.1186/s12913-019-4828-0 10.1186/s12913-019-4828-0 PMC6921405 31852546

[26] National Health Regulatory Authority. Pricing Guideline: National Health Regulatory Authority (NHRA): Kingdom of Bahrain; 2017.

[27] Alkhuzaee F.S., Almalki H.M., Attar A.Y., Althubiani S.I., Almuallim W.A., Cheema E. (2016 Dec). Evaluating community pharmacists’ perspectives and practices concerning generic medicines substitution in Saudi Arabia: A cross-sectional study. Health Policy.

[28] Alrasheedy AA, Hassali MA, Wong ZY, Aljadhey H, AL-Tamimi SK, Saleem F. Pharmaceutical Policy in Saudi Arabia. In: Babar Z-D, editor. Pharmaceutical policy in countries with developing healthcare systems [Internet]. Cham, SWITZERLAND: Springer International Publishing AG; 2017. Available from: http://ebookcentral.proquest.com/lib/londonschoolecons/detail.action?docID=4831867

[29] Business Monitor International (BMI). Morocco: Pharmaceuticals & Healthcare Report Q1 2017. Industry Report & Forecasts Series. London (UK); 2016.

[30] Kanavos P, Saleh S, Kamphuis BW, McClain K, El-Arnout N. Mapping of the Regulatory Environments in the Pharmaceutical Sector in 4 Middle East and North African Countries (unpublished work).

[31] Khan TM, Emeka P, Suleiman AK, Alnutafy FS, Aljadhey H. Pharmaceutical pricing policies and procedures in Saudi Arabia: A narrative review. Ther Innov Regul Sci [Internet]. 2015 Oct 26;50(2):236–40. Available from: https://doi.org/10.1177/2168479015609648 10.1177/2168479015609648 30227009

[32] Mohamed OE. The impact of direct price controls on pharmaceutical prices in Egypt. The University of Wisconsin - Madison: Ann Arbor.; 2014.

[33] O, Kreling DH. The impact of a pricing policy change on retail prices of medicines in Egypt. Value Heal Reg Issues [Internet]. 2016;10:14–8. Available from: https://www.sciencedirect.com/science/article/pii/S2212109916300231 10.1016/j.vhri.2016.04.004 27881272

[34] Saudi Food & Drug Authority. Pricing rules for pharmaceutical products. Saudi Food & Drug Authority; 2021.

[35] Wanis H. Pharmaceutical pricing in Egypt. In: Babar Z-D, editor. Pharmaceutical prices in the 21st century [internet]. Cham, Switzerland: Springer International Publishing AG; 2014. Available from: http://ebookcentral.proquest.com/lib/londonschoolecons/detail.action?docID=1965186

[36] Abuelkhair M., Abdu S., Godman B., Fahmy S., Malmström R.E., Gustafsson L.L. (2012 Feb). Imperative to consider multiple initiatives to maximize prescribing efficiency from generic availability: case history from Abu Dhabi. Expert Rev Pharmacoecon Outcomes Res.

[37] Al-Abbadi I., Qawwas A., Jaafreh M., Abosamen T., Saket M. (2009 Jun). One-year assessment of joint procurement of pharmaceuticals in the public health sector in Jordan. Clin Ther.

[38] World Health Organization, The Global Fund. Pharmaceutical sector country profile questionnaire: UAE; 2012.

[39] World Health Organization, The Global Fund. Pharmaceutical sector country profile questionnaire: OMAN; 2011.

[40] World Health Organization. Oman: pharmaceutical country profile; 2011.

[41] Awaisu A., Kheir N., Ibrahim M.I.M., El-Hajj M., Hazi H., Khudair N. (2014 Apr). Knowledge, attitudes, and practices of community pharmacists on generic medicines in Qatar. Int J Clin Pharm.

[42] World Health Organization, The global fund. Pharmaceutical sector country profile questionnaire: Saudi Arabia; 2012.

[43] World Health Organization. Jordan: Medicine prices, availability, affordability and price components [Internet]; 2007. Available from: https://apps.who.int/iris/handle/10665/116533

[44] World Health Organization. Egypt: Pharmaceutical country profile [Internet]; 2011. Available from: https://www.who.int/medicines/areas/coordination/Egypt_PSCPNarrativeQuestionnaire_27112011.pdf

[45] World Health Organization. Jordan: Pharmaceutical country profile; 2011.

[46] World Health Organization. Medicine prices, availability, affordability and price components: A synthesis report of medicine price surveys undertaken in selected countries of the WHO Eastern Mediterranean Region [Internet]; 2006. Available from: https://apps.who.int/iris/handle/10665/116567

[47] World Health Organization, The global fund. Pharmaceutical sector country profile questionnaire: Kuwait; 2012.

[48] World Health Organization, The global fund. Pharmaceutical sector country profile questionnaire: Bahrain; 2012.

[49] Business Monitor International (BMI). Algeria: Pharmaceuticals & Healthcare Report Q1 2017. Industry Report & Forecasts Series. London (UK); 2016.

[50] El-Dahiyat F, Kayyali R. Evaluating patients’ perceptions regarding generic medicines in Jordan. J Pharm policy Pract [Internet]. 2013 Jun 13;6:3. Available from: https://pubmed.ncbi.nlm.nih.gov/24764538 10.1186/2052-3211-6-3 PMC3987061 24764538

[51] World Health Organization, The global fund. Pharmaceutical sector country profile questionnaire: QATAR; 2011.

[52] World Health Organization, The global fund. Pharmaceutical sector country profile questionnaire: Jordan; 2011.

[53] Saleh S., Abou Samra C., Jleilaty S., Constantin J., El Arnaout N., Dimassi H. (2017 Oct). Perceptions and behaviors of patients and pharmacists towards generic drug substitution in Lebanon. Int J Clin Pharm.

[54] El-Jardali F., Fadlallah R., Morsi R.Z., Hemadi N., Al-Gibbawi M., Haj M. (2017 Feb). Pharmacists’ views and reported practices in relation to a new generic drug substitution policy in Lebanon: a mixed methods study. Implement Sci.

[55] World Health Organization, The global fund. Pharmaceutical sector country profile questionnaire: Lebanon; 2012.

[56] Business Monitor International (BMI). Saudi Arabia: Pharmaceuticals & Healthcare Report Q1 2017. Industry Report & Forecasts Series. London (UK); 2016.

[57] World Health Organization. Technical discussion on Medicine prices and access to medicines in the Eastern Mediterranean Region [Internet]. 2008. Available from: WHO Regional Office for the Eastern Mediterranean, World Health Organization

[58] Ball D, Tisocki K, Al-Saffar N. Medicine prices in the State of Kuwait: Report of a survey on medicine prices in Kuwait; 2005.

[59] Driouchi A., Zouag N. (2012). Pharmaceutical patents and prices of generics in morocco and neighboring economies. J World Intellect Prop.

[60] World Health Organization. Kuwait: Medicine prices, availability, affordability and price components; 2009.

[61] Alefan Q, Amairi R, Tawalbeh S. Availability, prices and affordability of selected essential medicines in Jordan: a national survey. BMC Health Serv Res [Internet]. 2018;18(1):787. Available from: https://doi.org/10.1186/s12913-018-3593-9 10.1186/s12913-018-3593-9 PMC6194614 30340486

[62] Ibrahim MI. Generic medicines policy in Qatar. Generics Biosimilars Initiat J (GaBI Journal). 2015;4(1):8.

[63] Kanavos P, Tzouma V, Fontrier A-M, Kamphuis B, Colville Parkin G, Saleh S. Pharmaceutical pricing and reimbursement in the middle east and north Africa Region: A mapping of the current landscape and options for the future; 2018.

[64] Brahmi S.A., Zahra Z.F., Seddik Y., Afqir S. (2016). The problem of the cost of new oncologic therapies: what about Morocco?. Pan Afr Med J.

[65] Business Monitor International (BMI). Lebanon: Pharmaceuticals & Healthcare Report Q1 2017. Industry Report & Forecasts Series. London (UK); 2016.

